# Signatures of Six Autophagy‐Related Genes as Diagnostic Markers of Thyroid‐Associated Ophthalmopathy and Their Correlation With Immune Infiltration

**DOI:** 10.1002/iid3.70093

**Published:** 2024-12-11

**Authors:** Qintao Ma, Yuanping Hai, Jie Shen

**Affiliations:** ^1^ Department of Endocrinology and Metabolism Shunde Hospital, Southern Medical University (The First People's Hospital of Shunde) Foshan Guangdong China

**Keywords:** biomarker, CIBERSORT, diagnostic biomarker, immune infiltration, thyroid‐associated ophthalmopathy

## Abstract

**Background:**

Thyroid‐associated ophthalmopathy (TAO) is one of the most complex autoimmune diseases in endocrinology areas. Autophagy‐related genes may be involved in the pathophysiology of TAO. This study aims to reveal key genes associated with autophagy in the pathogenesis and the potential diagnostic markers for TAO.

**Methods:**

We obtained autophagy‐related differential genes (AR‐DEGs) and their expression in TAO patients and controls. Gene ontology analysis (GO) and Kyoto Encyclopedia of Genes and Genomes (KEGG) analysis were used to perform the enrichment analysis of AR‐DEGs. LASSO regression, support vector machine recursive feature elimination, and random forest were performed to screen for disease signature genes (DSGs), which were further validated in another independent validation dataset. We used the receiver operating characteristic for the evaluation of the diagnostic efficacy of DSGs and also established a nomogram. The relative proportion of immune infiltration was calculated using the CIBERSORT algorithm, and the relationship between the identified gene markers and the level of infiltrating immune cells was explored.

**Results:**

We identified 24 AR‐DEGs, which were primarily enriched in cellular catabolic regulation, autophagosome membrane, and ubiquitin protein ligase binding in GO analysis, while KEGG analysis highlighted autophagy as the main enriched pathway. Six DSGs were identified by three algorithms. They were validated in another independent validation dataset. The combined six‐gene model also showed good diagnostic efficacy (AUC = 0.948). We further plotted the nomogram with better diagnostic efficacy. Immuno‐infiltration analysis and correlation analysis demonstrated that six DSGs were significantly correlated with the infiltrating immune cells.

**Conclusions:**

We identified several biological processes and pathways for the enrichment of AR‐DEGs. Six DSGs were identified, which showed great potential to become critical molecules in the diagnosis of TAO, and these DSGs showed a correlation with infiltrating immune cells.

## 1 Introduction

Thyroid‐associated ophthalmopathy (TAO) is an autoimmune disease that affects 25%–30% of patients with Graves' disease. Patients present with protruding eyes, tearing, photophobia, retraction of the upper eyelids, diplopia, and even dysthyroid optic neuropathy (DON), which seriously impairs vision. Patients have impaired visual function and facial disfigurement, resulting in poor quality of life and socioeconomic status [1, 2]. Glucocorticoids (GCs) remain the mainstay of treatment for active TAO during the peak of inflammation. However, GCs are also linked to a high number of adverse effects as well as post‐treatment relapse in some patients [3]. The pathogenesis of TAO is still unclear, but studies have revealed that it is mainly related to genetic, environmental, and autoimmune factors, with autoimmune disorders playing a particularly important role. Intricate interactions between potentially pathogenic autoantigens and autoantibodies, as well as activation of autoimmune reactions, lead to the release of relevant inflammatory factors, resulting in inflammation of orbital tissues, abnormal proliferation of fibroblasts, and ultimately thickening of the extraocular muscles and accumulation of fat [4, 5].

Autophagy is the process by which eukaryotic cells use lysosomes to degrade their own cytoplasmic proteins and damaged organelles under the regulation of autophagy‐related genes (ARGs) [6]. Autophagy includes basal autophagy under physiological conditions and induced autophagy under stress conditions. The former is a cellular self‐protection mechanism that benefits cell growth and development, protects cells from metabolic stress and oxidative damage, and plays an important role in maintaining intracellular homeostasis and the synthesis, degradation, and recycling of cellular products. Excessive autophagy under stressful conditions may result in metabolic stress, degradation of cellular components, and even cause cell death [7]. Autophagy plays an important role in various physiological and pathological processes, including cellular homeostasis, ageing, inflammation, immunity, and tumorigenesis [8, 9]. Autophagy is associated with a variety of autoimmune diseases, such as systemic lupus erythematosus (SLE), rheumatoid arthritis (RA), Crohn's disease (Crohn's disease) and ankylosing spondylitis (AS) [10]. Studies have shown that activation of autophagy is also directly associated with the development of multiple ocular diseases, including TAO, and that components of the autophagic pathway are constitutively expressed at high levels in the eye, including in the cornea, lens, retina, and orbit [11]. Yoon et al. [12] found that the expression of ARGs was significantly elevated in retro‐orbital tissues and orbital fibroblasts (OFs) of TAO patients compared with normal controls. The progression of inflammation in retro‐orbital tissues plays a critical role in the pathogenesis of TAO. Inflammatory cytokines, such as interleukin‐1β, can trigger autophagy, exacerbating the inflammatory response in orbital tissues and further accelerating disease progression. This is evidenced by the increased expression of autophagy‐related proteins Beclin‐1 and ATG‐5, as well as the conversion of LC3‐I to LC3‐II [13]. In addition, adipogenesis is an important pathological feature of TAO. Autophagy is essential for adipogenesis, and deletion of the ATG7 gene can inhibit fat formation, exerting an anti‐obesity effect [14]. Furthermore, according to the research by Li et al., icariin can inhibit the differentiation of preadipocytes into mature adipocytes by restoring the LC3‐II/LC3‐I ratio; this effect is mediated through the inhibition of the AMPK/mTOR pathway [15]. Similarly, studies have shown that neferine can inhibit autophagy‐induced adipogenesis in the OFs of GO patients by upregulating Nrf2 [13].

**Figure 1:**
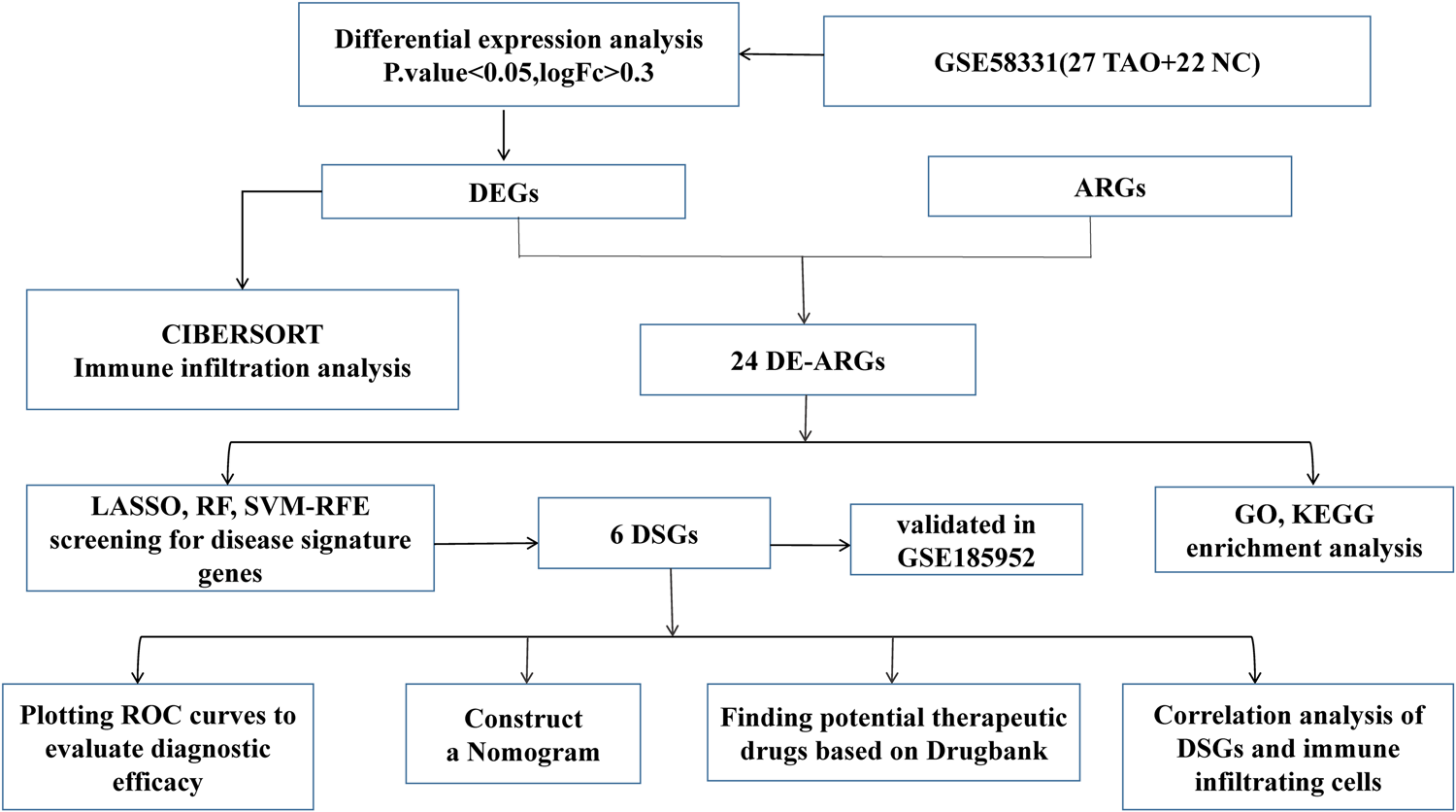
Flowchart of this study.

Therefore, this study sought to find feature genes based on autophagy process that may influence the diagnosis and treatment of TAO. In recent years, with the boom in bioinformatics and microarray technology, researchers have discovered genetic targets for many diseases by analyzing differentially expressed genes and potential signaling pathways. However, no study has explored the relationship between autophagy and TAO using bioinformatics analysis. The present study provides a preliminary exploration of the link between autophagy and TAO. The flow diagram of this study is shown in Figure 1.

## 2 Methods

### 2.1 Microarray Data Download and Autophagy‐Related Differential Genes (AR‐DEGs) Acquisition

We downloaded 222 autophagy‐relative genes (ARGs) from Human Autophagy Database (http://www.autophagy.lu/index.html). GEO is a high‐throughput resource functional genomics database including microarray and gene expression data. The GSE58331 dataset was obtained from the GEO database (http://www.ncbi.nlm.nih.gov/geo), with 27 and 22 orbital tissue samples from TAO patients and healthy individuals, respectively. The GSE185952 dataset was selected as an external validation set, which includes data on orbital adipose/connective tissue samples from TAO patients who underwent orbital decompression (*n* = 3), with control tissue obtained from the healthy people after plastic surgery (*n* = 3). Annotation information on the platform was used to convert the probes to gene symbols. We analyzed differentially expressed genes (DEGs) in retro‐orbital tissues of TAO patients and normal controls using the limma package with the criteria: *p* < 0.05 and |fold change (FC)| > 0.3. The obtained differentially expressed genes were intersected with ARGs, thus obtaining AR‐DEGs.

### 2.2 GO and KEGG Enrichment Analysis

The R‐package “clusterProfiler” was used to perform GO and KEGG enrichment analyses of AR‐DEGs to identify the potential molecular mechanisms of AR‐DEGs in TAO pathological processes. We further visualized the results using bubble diagram and histogram Map. To ensure the reliability of the enrichment results, we used Benjamini–Hochberg FDR to correct the *p*‐value for multiple hypothesis testing, and FDR < 0.05 indicated that the enrichment was statistically significant.

### 2.3 Identification of Disease‐Characteristic Genes

To obtain diagnostic markers for TAO, we used three machine learning algorithms (support vector machine recursive feature elimination [SVM‐RFE], least absolute shrinkage and selection operator [LASSO], and random forest [RF]) to filter the disease signature genes and take the intersection to derive the final diagnostic genes. These three algorithms were executed using the R packages “e1071,” “glmnet,” and “randomForest,” respectively. The LASSO regression algorithm uses regularization methods to improve the prediction accuracy. LASSO compresses the variable coefficients by constructing penalty functions so that some of the regression coefficients become zero, thus achieving the purpose of variable selection. RF is a compositional supervised learning method that is an extension of decision trees. This method constructs predictive models by sampling objects and variables, which means generating multiple decision trees and finally aggregating the classification results of each decision tree. Finally, based on the diagnostic genes obtained above, we plotted ROC curves to verify the accuracy of the diagnostic genes and used the area under curve (AUC) to assess the diagnostic effect of the genes. In addition, logistic regression was used to construct a multi‐gene prediction model.

### 2.4 Construction of the Nomogram Model

We used the R package “rms” to build a nomogram model based on selected disease‐characterizing genes. TAO is positively correlated with the total score. Finally, the predicted value of the occurrence of TAO in an individual was calculated by converting the relationship between the total score and the probability of TAO occurrence as a function of the total score. We tested the accuracy of the model by the analysis of calibration curves, decision curve analysis (DCA) curves, and clinical impact curves.

### 2.5 Immune Infiltration Analysis

To quantify the relative proportion of infiltrating immune cells in TAO gene expression profiles, we used a bioinformatics algorithm called CIBERSORT to calculate immune infiltration. The abundance of immune cells was estimated using a reference set of 22 immune cell subtypes (LM22) and 1000 alignments. Correlation analysis and visualization of 22 infiltrating immune cells was performed using the R package “corrplot.” Violin plots were drawn using the “vioplot” package in R to visualize the differences in immune infiltration between TAO and control samples. Correlations between the identified genetic biomarkers and the level of infiltrating immune cells were explored using Spearman's rank correlation analysis in R software. The obtained correlations were visualized using the graphical technique of the “ggplot2” package.

### 2.6 Search From DrugBank for Drugs Targeting Disease‐Characteristic Genes

We used the DrugBank database to predict drugs that may target diagnostic molecules. DrugBank includes thousands of drug records, each of which includes all relevant information on chemical structure, pharmacological properties, indications, toxicities, metabolic pathways, absorption rates, distribution rates, metabolic kinetics, drug interactions, etc. DrugBank, as a database with comprehensive drug information, can be widely used for bioinformatics analysis.

## 3 Results

### 3.1 Identification of Autophagy‐Related DEGs Associated With TAO

**Figure 2:**
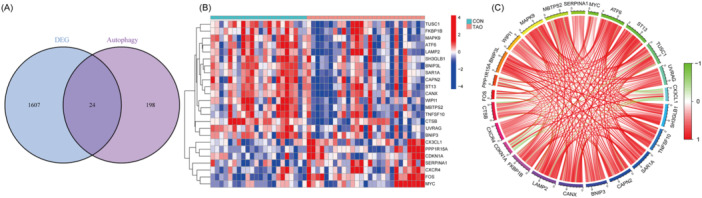
(A) Venn diagram showing the 24 DE‐ARGs obtained by intersecting the autophagy‐associated genes with the differential genes. (B) Heat map showing the differential expression of the 24 DE‐ARGs in the TAO and normal groups. (C) The circle diagram shows the correlation between 24 DE‐ARGs.

After setting the threshold (log2|FC| > 0.3, *p* < 0.05), we identified a total of 1631 DEGs. As the Venn diagram shows (Figure 2A), we then obtained 24 differentially expressed autophagy‐related genes (DE‐ARGs) after taking the intersection of the obtained DEGs with 222 ARGs. Of the DE‐ARGs we selected, 7 DEGs were expressed upregulated in TAO samples and 17 were expressed downregulated (Table S1). The heat map illustrates the difference in expression of DE‐ARGs between TAO and control groups (Figure 2B). The circle plot shows the correlation of expression between these 24 DE‐ARGs (Figure 2C).

### 3.2 Enrichment Analysis of DE‐ARGs

**Figure 3:**
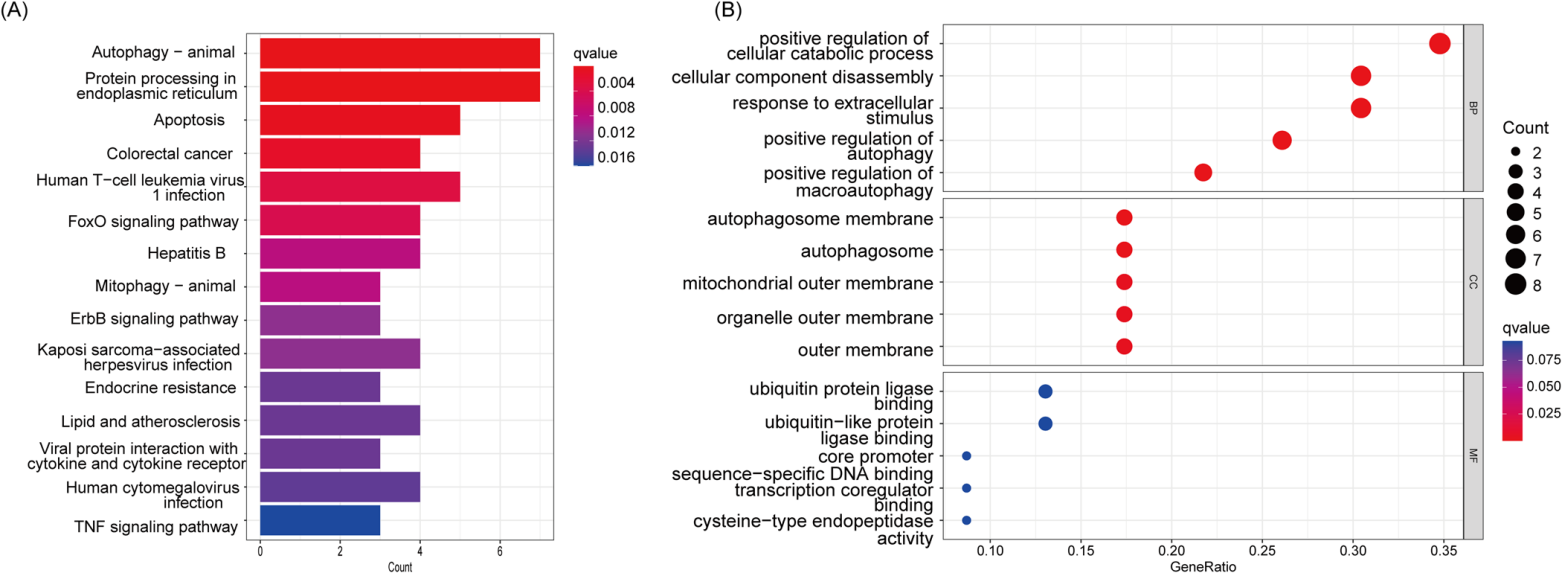
(A) KEGG enrichment analysis was performed on 24 DE‐ARGs and the results were presented as a histogram Map. (B) GO enrichment analysis of 24 DE‐ARGs, including BPs, CCs, and MFs, was performed and presented as a bubble plot.

AR‐DEGs were further analyzed through Gene Ontology and KEGG pathway analyses to explore the possible functions and pathways in the pathogenesis of TAO. In the KEGG analysis (Figure 3A), in addition to autophagy‐related pathways, significant enrichment was observed in pathways such as protein processing in the endoplasmic reticulum, apoptosis, and the FoxO signaling pathway. These pathways suggest that autophagy may be closely linked to cellular stress responses, protein folding, and apoptosis. Furthermore, the enrichment of the tumor necrosis factor (TNF) signaling pathway indicates a potential interaction between autophagy and inflammatory processes. Further GO enrichment analysis (Figure 3B) revealed that ARGs are localized in cellular components such as the organelles outer membrane and the autophagosome membrane, highlighting the structural role of autophagy within the cell. The GO analysis also pointed out that these genes are involved in biological processes such as response to extracellular stimulus and positive regulation of autophagy, further supporting the essential function of autophagy in TAO. Additionally, the enrichment of molecular functions such as ubiquitin protein ligase binding suggests that autophagy may play a role in regulating protein degradation and quality control.

### 3.3 Six Diagnostic AR‐DEGs Were Identified for TAO

**Figure 4:**
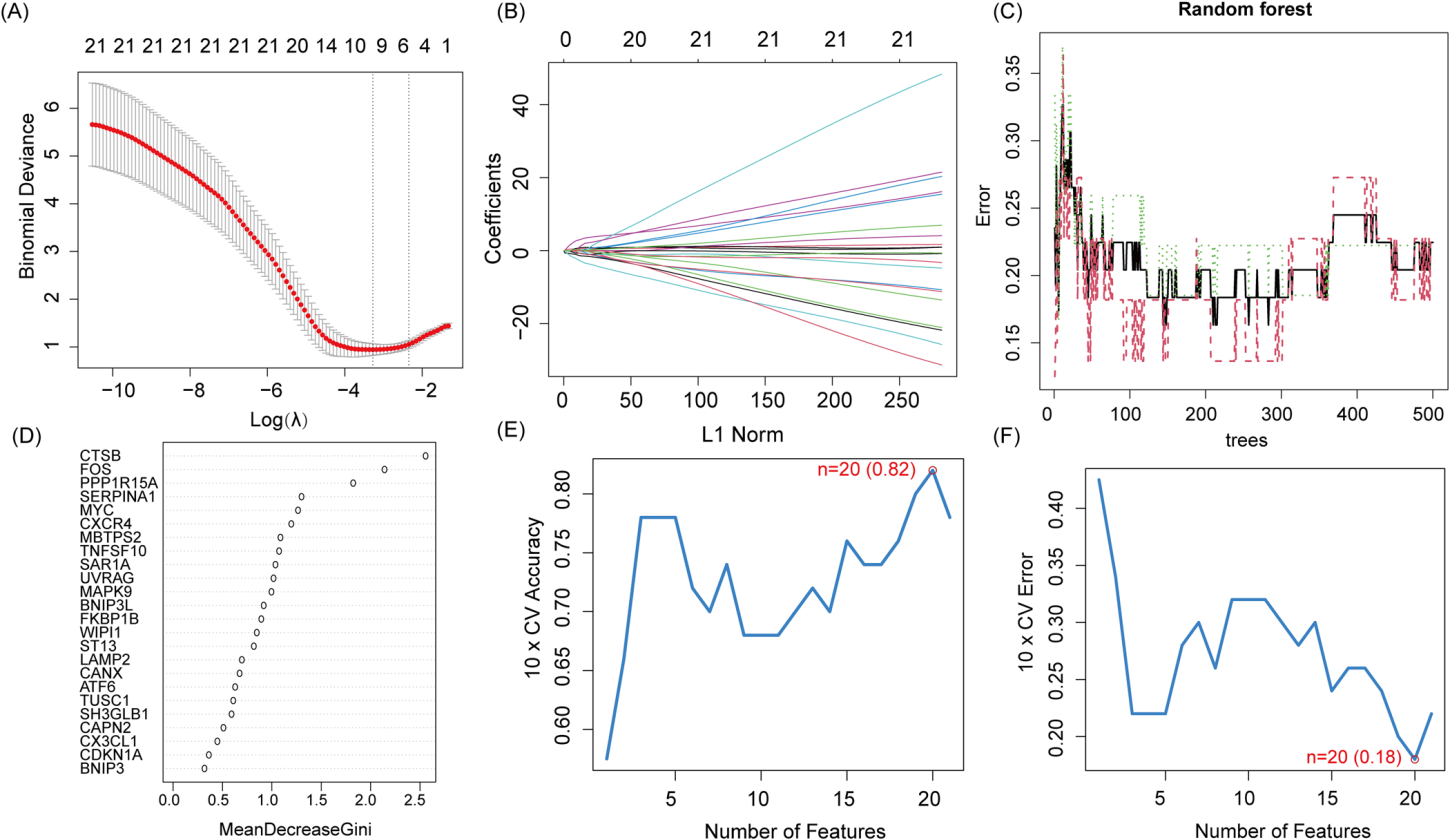
Six DSGs were identified as diagnostic genes for TAO. (A and B) DSGs were selected by the LASSO logistic regression algorithm with penalized parameter adjustment by 10‐fold cross‐validation. (C and D) The random forest shows the error of the AVC algorithm; genes are ranked according to significance scores. (E and F) The SVM‐RFE algorithm was used to filter the DSGs to identify the best combination of feature genes.

**Figure 5:**
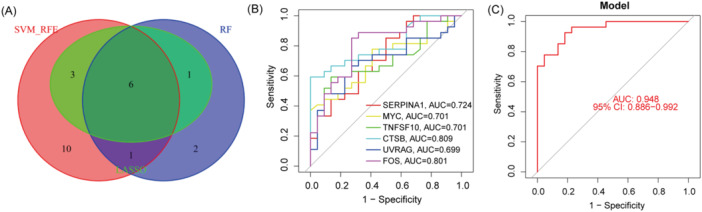
(A) Six DSGs obtained from the LASSO, RF, and SVM‐RFE models. (B) ROC curves for the six DSGs. (C) Logistic regression model to identify the AUC of DSGs.

Next, we performed three distinct machine learning algorithms (Figure 4) including LASSO, SVM‐RFE, and RF, to screen the significant DE‐ARGs to distinguish TAO from normal people. The genes screened by these three algorithms were summarized in Table S2. Then, we took the intersection of these three gene sets to obtain 6 disease signature genes (DSGs) including SERPINA1, MYC, TNFSF10, CTSB, UVRAG and FOS for subsequent analysis (Figure 5A). Moreover, to elucidate the ability of individual genes in distinguishing TAO from normal samples, ROC curves were generated for the 6 marker genes. The AUC for all genes was summary in Figure 5B. Finally, the ROC curve (Figure 5C) of diagnostic models generated from 6 DSGs show high diagnostic efficacy (AUC = 0.948). In addition, the diagnostic effectiveness of 6 DSGs was further validated in another independent dataset (GSE185952) (Figure S1).

### 3.4 Construction of the Nomogram Model

**Figure 6:**
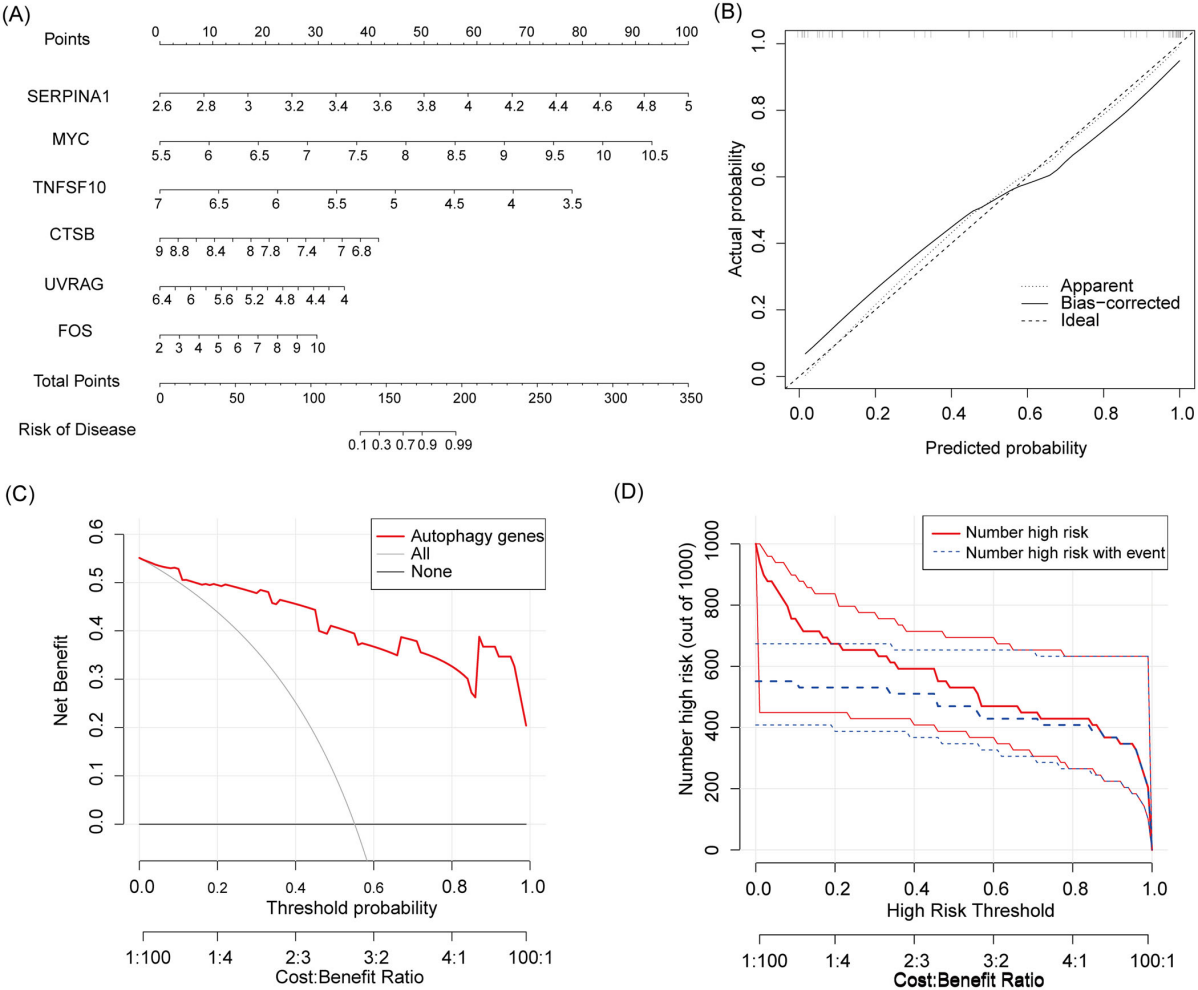
(A) The nomogram of six DSGs to predict the occurrence of TAO. (B) Calibration curves that can be used to assess the predictive power of nomogram. (C) DCA curves used to evaluate the clinical application value of the nomogram model. (D) Clinical impact curves of the nomogram model.

We constructed a nomogram model for six DSGs using logistic regression (Figure 6A). The predictivity of the new model was shown to be accurate through the use of calibration curves (the calibration curve [Figure 6B] shows a small error between the actual and predicted risk of TAO). As shown in Figure 6C, it may be deduced that judgments made by the use of the nomogram model may be beneficial to TAO patients. We also plotted the clinical impact curve (Figure 6D) based on the DCA curve. From 0 to 1, the “Number high risk” curve with a high‐risk threshold is very close to the “Number high risk with event” curve, indicating that the line graph model has a more accurate prediction ability.

### 3.5 Results of Infiltration of Immune Cell Subtypes

**Figure 7:**
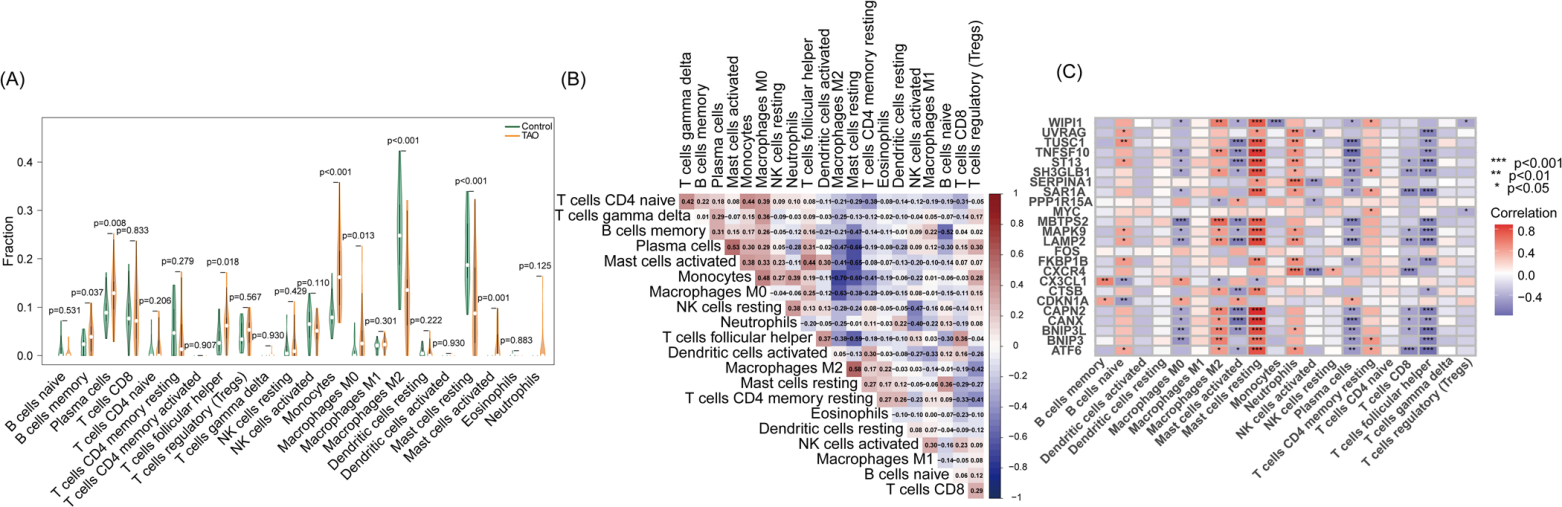
(A) Differences in immune infiltration between TAO patients and normal samples based on the CIBERSORT algorithm using violin plots. (B) Heatmap of immune cells correlation. (C) Pearson correlation analysis showing the correlation of 24 DE‐ARGs with infiltrated immune cells.

**Figure 8:**
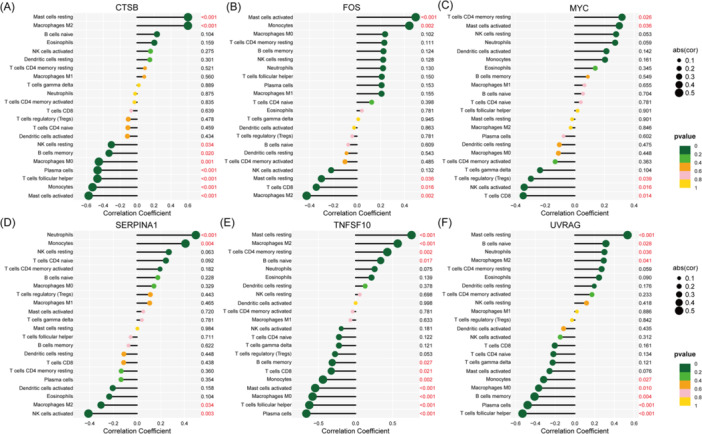
Demonstrate the correlation between six DSGs [including CTSB (A), FOS (B), MYC (C), SERPINA1 (D), TNFSF10 (E), and UVRAG (F)] and infiltrating immune cells.

The postorbital infiltration of immune cells in TAO patients has a crucial role in driving disease progression. First, we explored the composition of immune cells in TAO versus control orbital tissues and visualized the results using a violin plot (Figure 7A). In the TAO group, M2 Macrophages (*p* < 0.001), resting Mast cells (*p* < 0.001) showed lower levels of infiltration, while memory B cells (*p* = 0.037), Plasma cells (*p* = 0.008), T follicular helper cells (*p* = 0.018), Monocytes (*p* < 0.001), M0 Macrophages (*p* = 0.013), activated Mast cells (*p* = 0.001) showed higher levels of infiltration. In addition, we also used a heat map to present the correlation between the infiltration of different immune cells (Figure 7B). We then analyzed the correlation of six DSGs with infiltrating immune cells, as shown in Figure 8. FOS was positively correlated with activated Mast cells, Monocytes, and negatively correlated with resting Mast cells, CD8 ^+^T cells and M2 Macrophages. MYC was positively correlated with resting memory CD4^+^ T cells, activated Mast cells and negatively correlated with Tregs, activated NK cells, CD8^+^ T cells. SERPINA1 was positively correlated with Neutrophils, Monocytes and negatively correlated with M2 Macrophages, activated NK cells. TNFSF10 was positively correlated with resting Mast cells, Macrophages M2, resting memory CD4^+^ T cells, naive B cells, and negatively correlated with memory B cells, CD8^+^ T cells, Monocytes, activated Mast cells. M0 Macrophages, T follicular helper cells, and Plasma cells were negatively correlated. UVRAG was positively correlated with resting Mast cells, naïve B cells, Neutrophils, M2 Macrophages, and negatively correlated with Monocytes, M0 Macrophages, memory B cells, Plasma cells, T follicular helper cells. These results suggest that 6 DSGs were significantly correlated with infiltration of retro‐orbital immune cells. These ARGs may play an important role in the immune microenvironment of the retro‐orbital tissues of TAO patients.

### 3.6 Drug Results for These Disease Signature Genes

**Figure 9:**
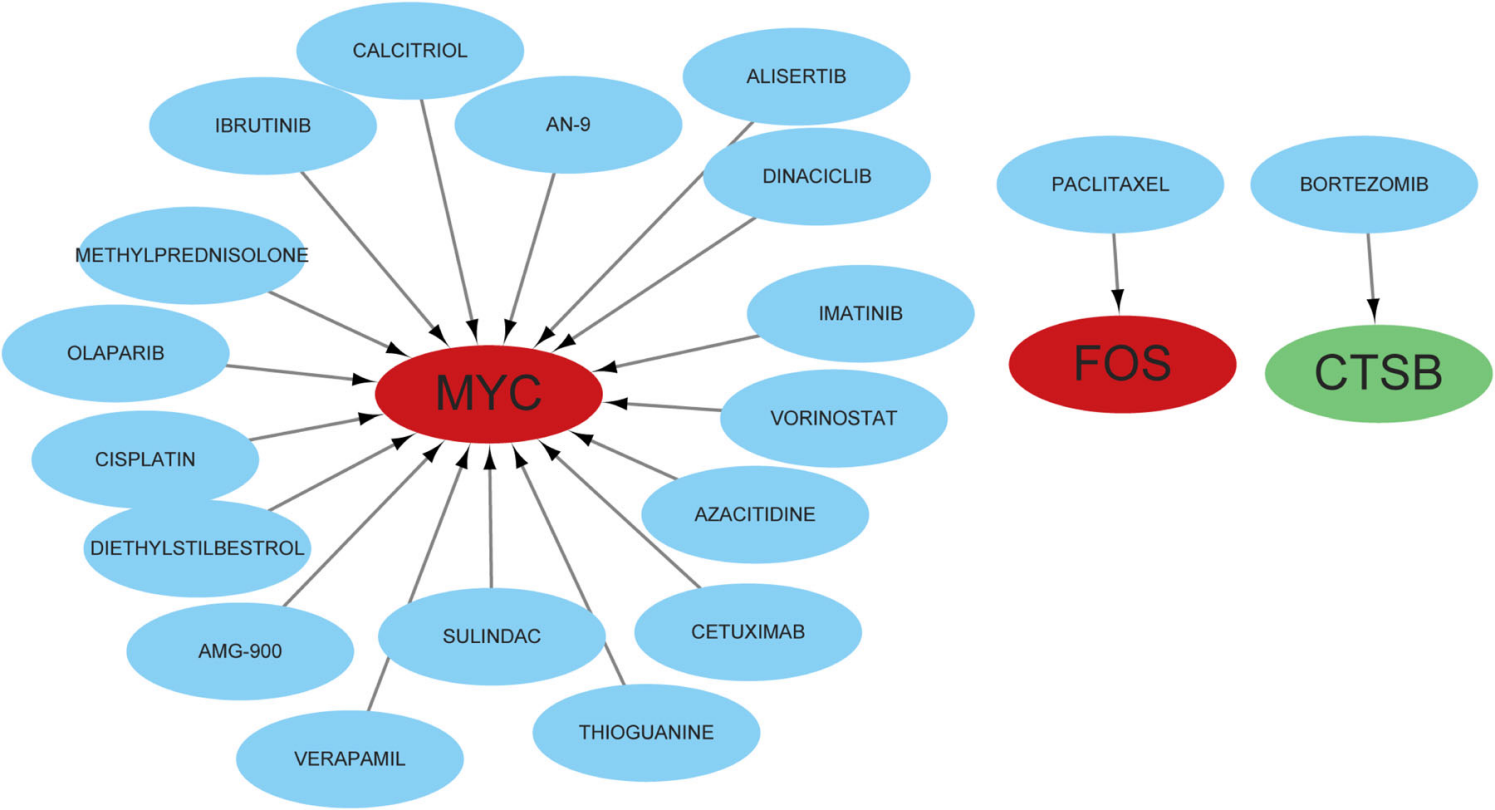
Search results from DrugBank for drugs targeting six DSGs.

Potential drugs targeting diagnostic molecules were predicted through the DrugBank database, and the results obtained were visualized by Figure 9. Paclitaxel was identified as a potential drug targeting FOS while bortezomib was identified as a potential drug targeting CTSB. Drugs targeting MYC include calcitriol, an‐9, alisertib, dinaciclib, imatinib, diethylstilbestrol, vorinostat, azacitidine, cetuximab, thioguanine, etc.

## 4 Discussion

In this current study, based on the Human Autophagy Database and the Gene Expression Omnibus Database, the differentially expressed genes between TAO patients and controls were first screened by R using the gene expression profile GSE58331, and then 24 AR‐DEGs including 7 upregulated and 17 downregulated genes were obtained by taking intersections with ARGs. GO and KEGG enrichment analysis showed that these genes were closely associated with signaling pathways such as autophagy, protein processing in endoplasmic reticulum, apoptosis, etc. From the results of BP, CC, MF analysis, we found that ARGs were enriched in positive regulation of cellular catabolic process, autophagosome membrane and ubiquitin protein ligase Binding, respectively. Furthermore, we identified six key biomarkers (SERPINA1, MYC, TNFSF10, CTSB, UVRAG and FOS) of TAO by SVM‐RFE, LASSO and RF. Among these, CTSB and FOS, as two ARGs which had the highest diagnostic effect, deserve to be further explored for their potential role in TAO pathogenesis. Notably, FOS, SERPINA1, and TNFSF10 also demonstrated strong diagnostic efficacy in the independent validation dataset. We also identified differences in infiltration of different types of immune cells in the TAO group versus normal controls retro‐orbital by immune infiltration analysis, and further elucidated the correlation between six disease signature genes and immune cells.

As a class of nuclear protein transcription factors, FOS has an important role in regulating cell growth, division, proliferation, differentiation, and even programmed death [16, 17, 18]. In addition, it has been shown that there is a very strong link between FOS and autophagy [19]. The association between FOS and TAO has also been reported. The expression of FOS in the extraocular muscles was significantly higher in the TAO group than in the control group, which is consistent with our results [20]. Studies have shown that FOS may act as a regulatory molecule in angiogenesis and endothelial cell inflammatory response [21, 22]. Our study shows that FOS gene expression correlates with infiltration of different immune cells. Infiltration of M2 macrophages was negatively correlated with FOS expression while monocyte infiltration was positively correlated with it.

Cathepsin B (CTSB) is one of the most abundant histone enzymes in the cytosol, usually in the form of inactive zymogens, and has a dual role as an endopeptidase and dipeptidase, and plays an important role in proteolytic metabolism [23]. Cathepsin B (CTSB) is responsible for driving the hydrolytic degradation of proteins in the lysosomal and extra‐lysosomal environment and play an essential role in autophagy [24]. It mediates the degradation of extracellular matrix proteins and the induction of apoptosis in tissues and cells, and also plays an important role in immune diseases such as AIDS and rheumatic. But its role in the pathogenesis of TAO remains unclear [25, 26]. In this current study, we found that CTSB was positively correlated with resting mast cells and M2 macrophages.

Serpin family A member 1 Gene (SERPINA1), encodes a protein that is a serine protease inhibitor whose targets include elastase, fibrin, thrombin, trypsin, pancreatic rennin and fibrinogen activator [27]. Recent studies have shown that SERPINA1 is closely associated with a variety of tumor diseases and autoimmune diseases such as ulcerative colitis and ANCA‐associated vasculitis [28, 29, 30]. Feng et al. [31] discovered that variants of SERPINA1 (such as the Z variant) accumulate in cells, increasing toxicity. SYVN1/HRD1 facilitates the degradation of these variants through SQSTM1/p62‐dependent autophagy, thereby reducing their toxic effects. When autophagy is impaired, SERPINA1 variants fail to degrade properly, leading to increased apoptosis [31]. A similar mechanism may be present in TAO, where upregulated SERPINA1 could regulate protein degradation via autophagy, impacting fibroblast and immune cell functions. Therefore, further research into the role of SERPINA1 in autophagy in TAO is crucial for advancing diagnosis and treatment strategies.

MYC is a broad‐ranging transcription factor that regulates cell differentiation and proliferation through a variety of mechanisms, including transcriptional amplification of target genes. A single‐cell sequencing study showed that MYC gene expression was significantly higher in the retro‐orbital connective tissue of TAO patients compared to normal controls which is consistent with our study. It has been shown that MYC expression is also associated with many immune diseases, such as myasthenia gravis, sicca syndrome, psoriasis, etc [32]. Our study also showed a correlation between retro‐orbital MYC expression and infiltrating immune cells in TAO patients. MYC has been shown to coordinate T cell metabolic reprogramming, cell proliferation, functional differentiation and apoptosis, resulting in a robust T cell‐mediated adaptive immune response [33]. MYC participates in autophagy formation and regulates autophagy through JNK1‐Bcl2 pathway and ROS. MYC inhibition leads to defects in autophagy formation, which leads to decreased autophagy [34].

UV radition resistance associated gene (UVRAG) encodes a protein that induces autophagy by interacting with BECLIN1. In this process, it promotes the binding of Atg14L and the formation of PI3K‐III complexes, which initiates the autophagic process [35]. It has also been reported that UVRAG dysfunction plays an important role in autoimmune diseases by affecting the process of autophagy. For example, abnormal autophagy in UVRAG knockout mice severely affects normal cell metabolism and also increases the risk of autoimmune diseases and malignancies in mice [36]. Our results tentatively suggest that UVRAG may play a potential role in the pathogenesis of TAO which was deserved to be studied further.

The protein encoded by TNFSF10 is a cytokine that belongs to the TNF ligand family. This protein preferentially induces apoptosis in transformed cells and tumor cells [37]. Studies have found that TNFSF10 can promote cellular autophagy [38]. Some recent studies have found that TNFSF10 may play an important role in some inflammation‐associated diseases [39, 40, 41]. In cutaneous melanoma, TNFSF10 is closely associated with autophagy and immune cell infiltration, with low expression being linked to poorer clinical outcomes [42]. However, no studies have yet directly explored the link between TNFSF10 and TAO. Whether there is an association between TNFSF10 and TAO and its mechanism of action can be further investigated.

In this work, we analyzed the potential role of autophagy genes in the pathogenesis of TAO based on bioinformatics principles and investigated the correlation between ARGs and postorbital immune infiltration, and provided diagnostic models and therapeutic agents based on six DSGs. However, there are some limitations of this study. First, the relatively small sample size of the validation dataset may impact the statistical power and somewhat limit the generalizability of the findings. Therefore, we recommend that future studies validate these results in larger cohorts to enhance the reliability and robustness of the conclusions. Second, the gene studies in our study were limited to the transcriptional level. Future studies should incorporate these aspects to provide a more comprehensive understanding. Lastly, although our results suggest associations between ARGs and TAO, there is still a lack of studies addressing the specific roles of these genes in TAO. We strongly recommend functional validation of these genes through in vitro and in vivo experiments to elucidate their precise molecular mechanisms in TAO pathogenesis.

## 5 Conclusion

we identified several biological processes and pathways for the enrichment of AR‐DEGs. Six DSGs were identified which showed great potential to become key molecules in the diagnosis of TAO and these DSGs showed correlation with infiltrating immune cells. Our findings provide new insights into the association of ARGs with TAO. Our findings provide new insights into the association of ARGs with TAO. Future in vitro and in vivo validation is expected to further confirm our findings.

## Author Contributions

Conceptualization: Jie Shen and Yuanping Hai. Methodology and formal analysis: Qintao Ma. Resources: Jie Shen. Data curation: Yuanping Hai. Writing—original draft preparation: Qintao Ma. Writing—review and editing: Yuanping Hai and Jie Shen. Supervision: Jie Shen and Yuanping Hai. Project administration: Jie Shen and Yuanping Hai.

## Ethics Statement

The authors have nothing to report.

## Consent

The authors have nothing to report.

## Conflicts of Interest

The authors declare no conflicts of interest.

## Supporting information


**Supplementary Table 1.** Differences in expression of 24 AR‐DEGs between TAO and control groups.

**Supplementary Table 2.** 6 DSGs obtained by three different machine learning algorithms (SVM‐RFE, RF and LASSO).

**Supplementary figure1.** The diagnostic effectiveness of 6 DSGs was further validated in the independent dataset (GSE185952).

## Data Availability

The datasets of the current study are available in the Gene Expression Omnibus (GEO) datasets (http://www.ncbi.nlm.nih.gov/geo/) and Human Autophagy Database (http://www.autophagy.lu/index.html).
