# Supplementary figures and images for: Signatures of Six Autophagy‐Related Genes as Diagnostic Markers of Thyroid‐Associated Ophthalmopathy and Their Correlation With Immune Infiltration
Source: Immun Inflamm Dis. 2024 Dec 11;12(12):e70093. doi: 10.1002/iid3.70093 (PMC11633049; doi:10.1002/iid3.70093)

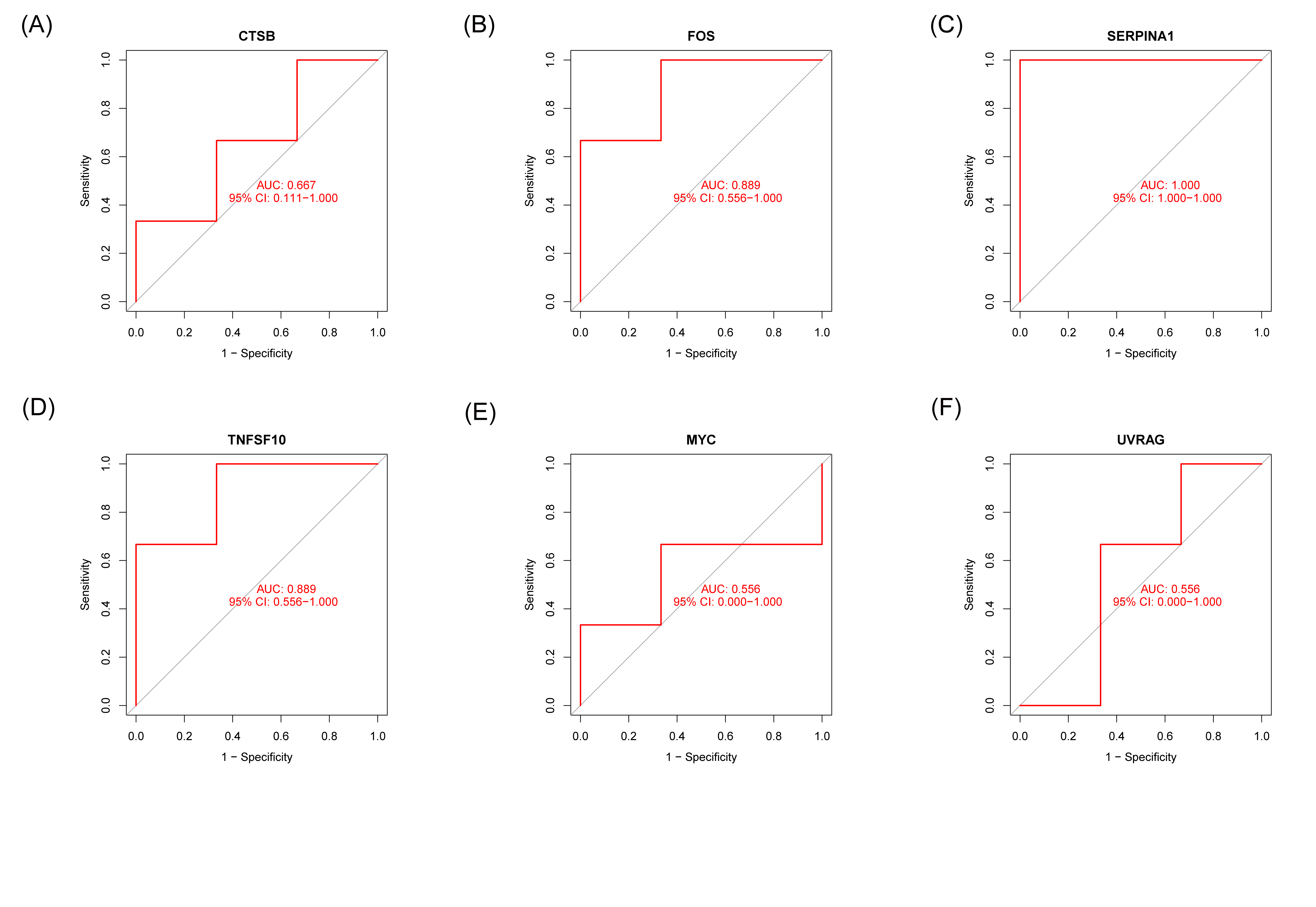

Supplement: Supplementary file 3 — Supplementary figure1. The diagnostic effectiveness of 6 DSGs was further validated in the independent dataset (GSE185952). [file IID3-12-e70093-s002.tif]
